# Clip and cure: A minimally invasive strategy for left atrial appendage exclusion and left atrial cryoablation lesion set

**DOI:** 10.1016/j.xjtc.2026.102329

**Published:** 2026-03-24

**Authors:** Abdullatif Abo Dan, Nabil Ajmi, Philippe Demers, Michel Pellerin, Denis Bouchard

**Affiliations:** Department of Cardiac Surgery, Montreal Heart Institute and Université de Montréal, Montreal, Québec, Canada

**Keywords:** left atrial appendage exclusion, surgical clip, left-sided cryo lesion set, minithoracotomy

## Abstract

**Objectives:**

Surgical exclusion of the left atrial appendage (LAA) combined with left atrial cryoablation is an important component of atrial fibrillation management during mitral valve surgery. A right minithoracotomy approach offers direct access to the transverse and oblique sinuses, enabling controlled LAA clip deployment and delivery of cryoablation lesions.

**Methods:**

A video-assisted right minithoracotomy was performed through the fourth intercostal space. Intraoperative transesophageal echocardiography guided LAA sizing with a 10-mm safety margin and confirmed adequate distance from the circumflex artery and warfarin ridge. After initiation of cardiopulmonary bypass, the LAA was mobilized and excluded using a preselected clip introduced through the transverse sinus under direct visualization, avoiding adjacent structures. A left atrial cryoablation lesion set was then performed, including 4 components: endocardial application toward the mitral annulus, epicardial convergence across the coronary sinus, and sequential roof and inferior pulmonary vein lines completing a box lesion pattern. Continuous probe contact with controlled −60 °C was used to achieve transmural lesions.

**Results:**

The minimally invasive approach provided excellent visualization of the transverse sinus, allowing precise LAA clip placement and secure appendage exclusion. The cryoablation sequence facilitated the creation of contiguous and transmural lesion lines, completing a comprehensive left atrial lesion set through a limited thoracotomy.

**Conclusions:**

A minimally invasive right minithoracotomy technique allows reliable LAA exclusion combined with left atrial cryoablation during mitral valve surgery. Direct visualization through the transverse sinus supports accurate clip deployment, whereas a structured cryoablation sequence enables consistent lesion formation for the surgical treatment of atrial fibrillation.

**Figure:**
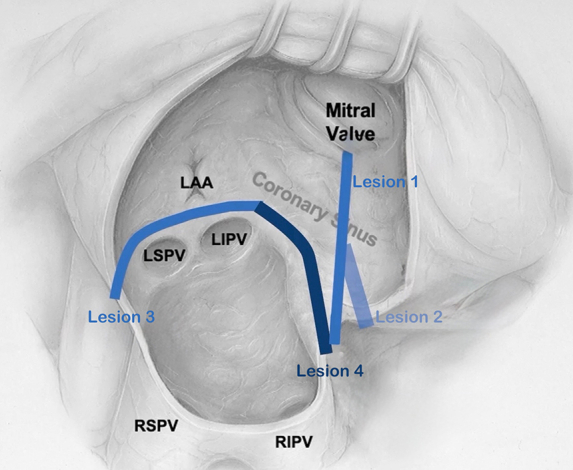
Schematic representation of left atrial cryoablation lesion sets with exclusion of the left atrial appendage.

**Central Message**

A minimally invasive approach allows controlled LAA clip closure through the transverse sinus and complete left atrial cryoablation with excellent exposure and procedural precision.

**Perspective**

A minimally invasive approach allows controlled LAA clip closure through the transverse sinus and complete left atrial cryoablation with excellent exposure and procedural precision.

Surgical exclusion of the left atrial appendage (LAA) and left-sided cryoablation lesion set are key components of contemporary management of atrial fibrillation, particularly in patients undergoing concomitant mitral surgery.^1^^,^^2^ Minimally invasive approaches through a right minithoracotomy have evolved to allow precise access to the transverse and oblique sinuses, enabling safe placement of the LAA clip and controlled left-atrial cryoablation.^3^ Video 1 illustrates a systematic technique for LAA exclusion using a clip device combined with a structured left-sided cryoablation lesion set via a minimitral incision.

## Methods and Procedures

The right minithoracotomy, initially about 3 to 4 cm and slightly stretched with soft tissue retractors, was performed through the fourth intercostal space, with ports placed for the camera (third intercostal space), cardioplegia probe, suction, carbon dioxide insufflation, and aortic clamp. The institutional review board or equivalent ethics committee of the Montreal Heart Institute did not approve this study because it is an anonymous technical video and not a cohort study. The requirement for patient informed consent was waived due to the anonymized and educational nature of this video case submission.

This technique has been applied in 85 consecutive patients at our center since 2023 for combined minimally invasive mitral valve surgery with concomitant left-sided cryoablation and LAA clipping. Age and reduced left ventricular ejection fraction alone were not exclusion criteria, in accordance with guideline recommendations supporting concomitant atrial fibrillation ablation during mitral surgery. Successful ligation was defined as complete exclusion on intraoperative transesophageal echocardiography (TEE) with no residual stump >5 mm and no residual Doppler flow. By this definition, immediate intraoperative ligation success was achieved in all cases.

Preoperative high-resolution computed tomography is recommended to evaluate LAA morphology and assist in clip size planning, with correlation to intraoperative TEE measurements. In this procedure, we began with precise sizing of the LAA using intraoperative TEE. The maximum LAA ostial width measured 18 mm in the long-axis view. To ensure complete coverage, a 10-mm safety margin was added on each side, resulting in selection of a 40-mm clip. TEE also delineated the course of the left circumflex coronary artery and the warfarin ridge to confirm safe clip positioning and preservation of flow after deployment.

LAA exclusion was performed using the AtriClip Pro-V device (AtriCure), a thoracoscopic-optimized member of the AtriClip family with an extended articulated delivery shaft designed for minimally invasive access. Manufacturer contraindications include active systemic infection, bacterial endocarditis, or infected operative fields.

Redo operations with dense pericardial adhesions or extreme LAA anatomies not amenable to clip sizing may preclude safe deployment. Undersized clips risk incomplete exclusion, whereas oversized clips may compress adjacent structures such as the left upper pulmonary vein or circumflex artery. Therefore, availability of multiple clip sizes is recommended.

Limited visualization inherent to right minithoracotomy may challenge base identification, and gentle retraction is recommended. Typically, the evaluation of the access to the LAA takes place after cardiopulmonary bypass begins. In more challenging cases where exposure is compromised, the exploration is performed after aortic crossclamping, the delivery of cardioplegia, and decompression of the left atrium, thereby creating a spacious working field beneath the aorta. Through the transverse sinus, the LAA was gently mobilized toward the preselected clip using atraumatic forceps. Countertraction allowed advancement of the open clip toward the appendage base while maintaining close alignment to the left atrial wall. Then, the right lever was flexed to position the clip deeply at the base, ensuring no impingement on nearby structures, including the circumflex artery, pulmonary veins, or pulmonary artery. After confirming that 2 arms encompassed the appendage, the clip was closed, deployed, and the delivery system withdrawn from the chest, yielding complete LAA exclusion under direct visualization and intraoperative TEE confirmation, defined as absence of residual Doppler flow and a residual stump ≤5 mm. In 2 out of 85 cases, an initial clip position was judged unsatisfactory intraoperatively and a second clip was applied more proximally to achieve complete exclusion. Procedural efficiency improved after approximately 10 initial cases, with typical clip deployment times of 3 to 5 minutes thereafter.

The next step was cryoablation lesions of the left atrium. The left-sided lesion set includes pulmonary vein isolation, a mitral isthmus connection, a superior pulmonary vein-to-LAA base line, and a posterior left atrial epicardial line. This lesion configuration represents a left-sided cryoablation strategy inspired by contemporary left-sided CryoMaze techniques described by McCarthy and Cox, without routine right-atrial lesion creation.^4^ In a recent series, concomitant left-sided cryoablation during totally endoscopic mitral valve surgery demonstrated favorable long-term rhythm outcomes, with freedom from atrial fibrillation reported in approximately 96% at 1 year and 69% at 5 years.^5^

With full atrial exposure, the malleable cryoprobe, shaped to ensure continuous tissue contact, was applied endocardially from the inferior border of the left atriotomy toward the mitral annulus at −60 °C for 3 minutes. This first lesion requires special attention to the phrenic nerve to prevent impingement and damage. During the freeze cycle, valve inspection and annular suturing can be performed. The probe’s active defrost function allowed atraumatic release without saline irrigation. Suction of warm blood was used to maintain a dry field and preserve thermal integrity for a more efficient cryoablation. A second lesion was applied epicardially through the oblique sinus, overlapping the endocardial line over the coronary sinus while frosting of the first lesion remained visible. A third lesion was applied along the atrial roof and lateral aspect of the left pulmonary veins, extending from the left side of the atriotomy between the left pulmonary veins and the excluded LAA. A fourth lesion completed encirclement of the inferior pulmonary veins, connecting to form the base of the box lesion set. The probe was set at −60 °C and applied for 3 full minutes for all endocardial lesions and for 2 minutes for the epicardial lesion on the thin coronary sinus. The 3 endocardial cryoablation lines require approximately 9 minutes in total, with an additional 2 minutes for the epicardial posterior line. Modest additional crossclamp time for left-sided cryoablation was not considered a contraindication.

## Conclusions

This totally minimally invasive approach enables concomitant mitral valve surgery, reliable LAA exclusion, and a structured left-sided cryoablation lesion set inspired by cyroablation principles without routine right-atrial lesion creation. Direct visualization via the transverse sinus ensures accurate and reproducible LAA clip placement, whereas the described cryoablation sequence facilitates creation of contiguous transmural lesions intended to support rhythm control in selected patients. Centers adopting this strategy are encouraged to monitor crossclamp duration and procedural outcomes prospectively and to apply conservative patient selection during the initial learning curve. Long-term rhythm outcomes and equivalence to full biatrial Cox-maze strategies require further prospective evaluation, but this left-sided approach has shown favorable outcomes in recent studies.

### Webcast

You can watch a Webcast of this AATS meeting presentation by going to: https://www.aats.org/resources/left-atrial-appendage-exclusio-11387.

**Figure:**
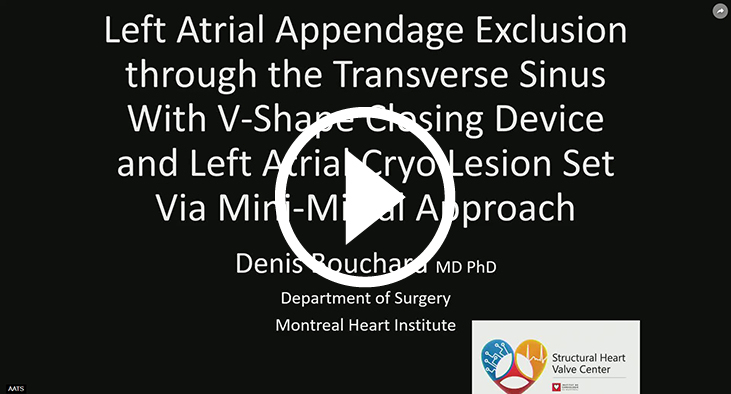

## Conflict of Interest Statement

Dr Bouchard has received consulting or proctoring fees from Edward Lifesciences and AtriCure. All other authors reported no conflicts of interest.

The *Journal* policy requires editors and reviewers to disclose conflicts of interest and to decline handling or reviewing manuscripts for which there may be a conflict of interest. The editors and reviewers of this article have no conflicts of interest.

## Supplementary Data

Video 1 This video demonstrates minimally invasive left atrial appendage (LAA) exclusion and left atrial cryoablation through a right minithoracotomy during mitral valve surgery. Key steps include intraoperative transesophageal echocardiography-guided LAA sizing, clip deployment through the transverse sinus, and completion of a left atrial cryoablation lesion set with mitral annular, coronary sinus, roof, and inferior pulmonary vein lines to create a box lesion pattern. Video available at: https://www.jtcvs.org/article/S2666-2507(26)00136-7/fulltext.
