# Supplementary figures and images for: Clip and cure: A minimally invasive strategy for left atrial appendage exclusion and left atrial cryoablation lesion set
Source: JTCVS Tech. 2026 Mar 24;37:102329. doi: 10.1016/j.xjtc.2026.102329 (PMC13261284; doi:10.1016/j.xjtc.2026.102329)

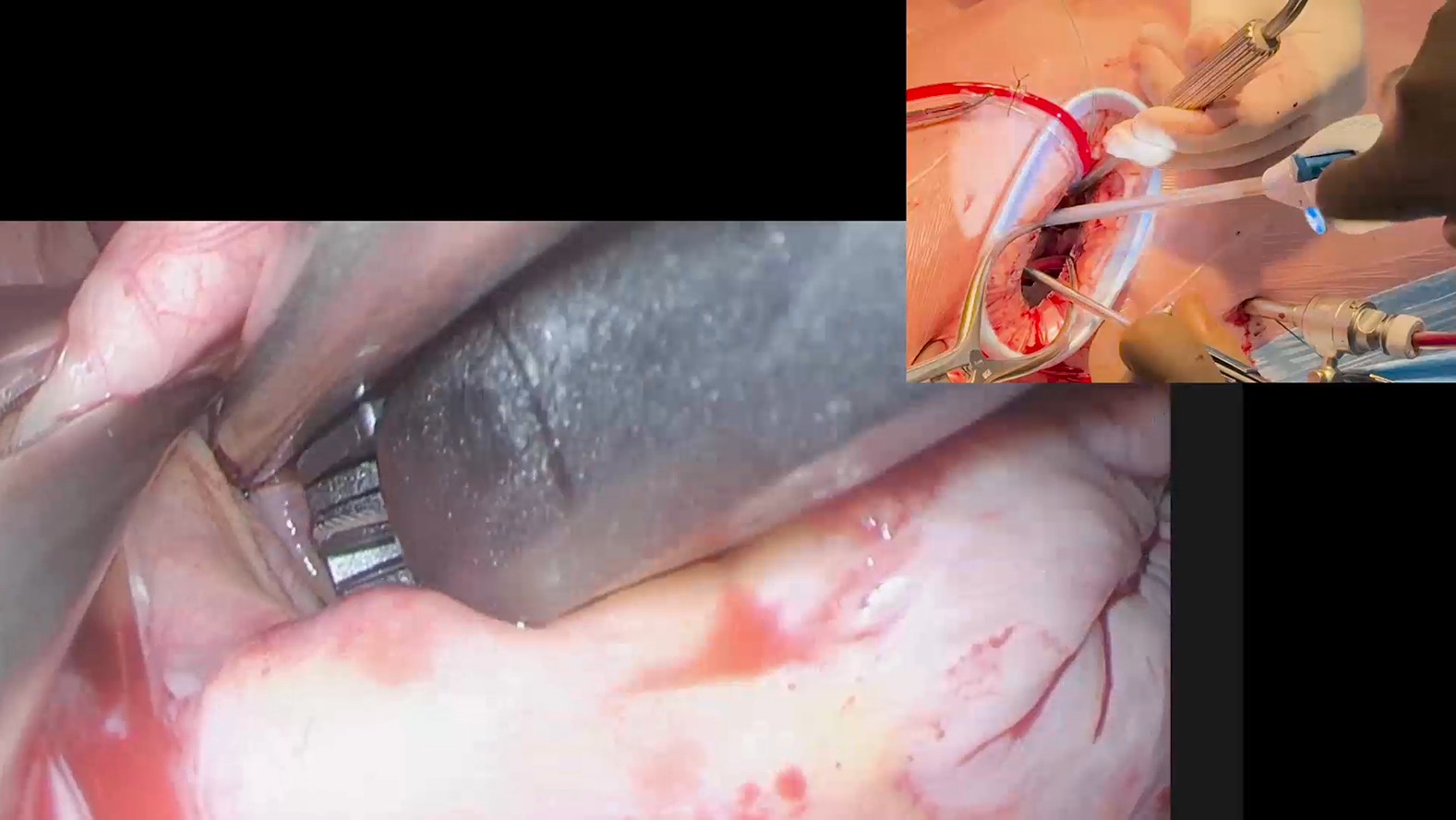

Supplement: Video 1 — This video demonstrates minimally invasive left atrial appendage (LAA) exclusion and left atrial cryoablation through a right minithoracotomy during mitral valve surgery. Key steps include intraoperative transesophageal echocardiography-guided LAA sizing, clip deployment through the transverse sinus, and completion of a left atrial cryoablation lesion set with mitral annular, coronary sinus, roof, and inferior pulmonary vein lines to create a box lesion pattern. Video available at: https://www.jtcvs.org/article/S2666-2507(26)00136-7/fulltext. [file fx3.jpg]
